# Supplementary material for: Empirical methods for controlling false positives and estimating confidence in ChIP-Seq peaks
Source: BMC Bioinformatics. 2008 Dec 5;9:523. doi: 10.1186/1471-2105-9-523 (PMC2628906; doi:10.1186/1471-2105-9-523)
Supplement: Additional File 1 — A variety of html documents from the USeq web site detailing the available applications, their best usage, output file type descriptions, command line menus, etc. [file 1471-2105-9-523-S1.zip › USeqUserGuides/JavaDoc.zip!/JavaDoc/trans/roc/MethodTester.html]

MethodTester


|  |  |  |
| --- | --- | --- |
| **Overview** \| **Package** \| **Class** \| **Use** \| **Tree** \| **Deprecated** \| **Index** \| **Help** | |  |
| **PREV CLASS**   **NEXT CLASS** | **FRAMES**    **NO FRAMES**     **All Classes** |  |
| SUMMARY: NESTED \| FIELD \| CONSTR \| METHOD | DETAIL: FIELD \| CONSTR \| METHOD |  |


---


## trans.roc Class MethodTester

```
java.lang.Object
  trans.roc.MethodTester
```

---

``` public class MethodTester extends java.lang.Object ```

Application for scoring '.sgr' files for positives and negatives in relation to known (spike in) data.

---

**Constructor Summary**

`MethodTester(java.lang.String[] args)`


| **Method Summary** | |
| --- | --- |
| `void` | `binSgrs()`             Runs through an array of Sgr[] noting whether it falls in the positive regions or not positive regions |
| `void` | `blankPositives()`             Resets the positive regions to their instantiation state. |
| `void` | `calculateBinFDRs()`             Runs through all the bins calculating and setting fdrs for each. |
| `Bin` | `findBin(Sgr sgr)`             Finds an appropriate bin given an sgr object |
| `static double` | `fractionPositive(java.util.ArrayList doubles, double scoreCutOff, double divider)`             Given an ArrayList of Double, calculates the fraction that is >= scoreCutOff. |
| `static void` | `main(java.lang.String[] args)` |
| `void` | `makeBins()` |
| `void` | `makePositives()` |
| `void` | `makePositives(java.io.File regions)` |
| `static Sgr[]` | `parseSgrFile(java.io.File sgrFile, boolean zeroNaNOrInfinityScores)`             Reads in an Sgr file. |
| `static int` | `positive(Positive[] pos, Sgr sgr)`             Checks to see if an sgr object is one of the positives, if not returns -1, otherwise returns index number. |
| `boolean` | `resetStartStop()`             Scans the bins to determine the optimal start and stop for the next round. |

**Methods inherited from class java.lang.Object**

`equals, getClass, hashCode, notify, notifyAll, toString, wait, wait, wait`

**Constructor Detail**

### MethodTester

```
public MethodTester(java.lang.String[] args)
```


**Method Detail**

### calculateBinFDRs

```
public void calculateBinFDRs()
```

:   Runs through all the bins calculating and setting fdrs for each.

---


### binSgrs

```
public void binSgrs()
```

:   Runs through an array of Sgr[] noting whether it falls in the positive regions or not positive regions

---


### positive

```
public static int positive(Positive[] pos,
                           Sgr sgr)
```

:   Checks to see if an sgr object is one of the positives, if not returns -1, otherwise returns index number.

---


### findBin

```
public Bin findBin(Sgr sgr)
```

:   Finds an appropriate bin given an sgr object

---


### main

```
public static void main(java.lang.String[] args)
```

---


### fractionPositive

```
public static double fractionPositive(java.util.ArrayList doubles,
                                      double scoreCutOff,
                                      double divider)
```

:   Given an ArrayList of Double, calculates the fraction that is >= scoreCutOff.

---


### makeBins

```
public void makeBins()
```

---


### makePositives

```
public void makePositives()
```

---


### blankPositives

```
public void blankPositives()
```

:   Resets the positive regions to their instantiation state.

---


### makePositives

```
public void makePositives(java.io.File regions)
```

---


### parseSgrFile

```
public static Sgr[] parseSgrFile(java.io.File sgrFile,
                                 boolean zeroNaNOrInfinityScores)
```

:   Reads in an Sgr file. Chrom, position, score.
    Returns null if not a number or infinity is found and the zero flag is false.

---


### resetStartStop

```
public boolean resetStartStop()
```

:   Scans the bins to determine the optimal start and stop for the next round.
    Returns true if start and stop were reset and another round should be run.
    Returns false if not reset.


---


|  |  |  |
| --- | --- | --- |
| **Overview** \| **Package** \| **Class** \| **Use** \| **Tree** \| **Deprecated** \| **Index** \| **Help** | |  |
| **PREV CLASS**   **NEXT CLASS** | **FRAMES**    **NO FRAMES**     **All Classes** |  |
| SUMMARY: NESTED \| FIELD \| CONSTR \| METHOD | DETAIL: FIELD \| CONSTR \| METHOD |  |


---
